# Reovirus σNS and μNS Proteins Remodel the Endoplasmic Reticulum to Build Replication Neo-Organelles

**DOI:** 10.1128/mBio.01253-18

**Published:** 2018-08-07

**Authors:** Raquel Tenorio, Isabel Fernández de Castro, Jonathan J. Knowlton, Paula F. Zamora, Christopher H. Lee, Bernardo A. Mainou, Terence S. Dermody, Cristina Risco

**Affiliations:** a Cell Structure Laboratory, National Center for Biotechnology, CNB-CSIC, Campus UAM, Madrid, Spain; b Department of Pathology, Microbiology, and Immunology, Vanderbilt University School of Medicine, Nashville, Tennessee, USA; c Department of Microbiology and Molecular Genetics, University of Pittsburgh School of Medicine, Pittsburgh, Pennsylvania, USA; d Department of Pediatrics, Vanderbilt University School of Medicine, Nashville, Tennessee, USA; e Department of Pediatrics, University of Pittsburgh School of Medicine, Pittsburgh, Pennsylvania, USA; Johns Hopkins Bloomberg School of Public Health

**Keywords:** endoplasmic reticulum, membrane remodeling, reovirus, virus factory biogenesis

## Abstract

Like most viruses that replicate in the cytoplasm, mammalian reoviruses assemble membranous neo-organelles called inclusions that serve as sites of viral genome replication and particle morphogenesis. Viral inclusion formation is essential for viral infection, but how these organelles form is not well understood. We investigated the biogenesis of reovirus inclusions. Correlative light and electron microscopy showed that endoplasmic reticulum (ER) membranes are in contact with nascent inclusions, which form by collections of membranous tubules and vesicles as revealed by electron tomography. ER markers and newly synthesized viral RNA are detected in inclusion internal membranes. Live-cell imaging showed that early in infection, the ER is transformed into thin cisternae that fragment into small tubules and vesicles. We discovered that ER tubulation and vesiculation are mediated by the reovirus σNS and μNS proteins, respectively. Our results enhance an understanding of how viruses remodel cellular compartments to build functional replication organelles.

## IMPORTANCE

Viruses modify cellular structures to build replication organelles. These organelles serve as sites of viral genome replication and particle morphogenesis and are essential for viral infection. However, how these organelles are constructed is not well understood. We found that the replication organelles of mammalian reoviruses are formed by collections of membranous tubules and vesicles derived from extensive remodeling of the peripheral endoplasmic reticulum (ER). We also observed that ER tubulation and vesiculation are triggered by the reovirus σNS and μNS proteins, respectively. Our results enhance an understanding of how viruses remodel cellular compartments to build functional replication organelles and provide functions for two enigmatic reovirus replication proteins. Most importantly, this research uncovers a new mechanism by which viruses form factories for particle assembly.

## INTRODUCTION

Cell membranes function as platforms to coordinate numerous steps in viral replication (1, 2). Mitochondria, lysosomes, phagosomes, Golgi complex, peroxisomes, and endoplasmic reticulum (ER) are subverted and remodeled by viruses (3, 4), many of which use the ER as a preferred membranous compartment to build replication organelles (5).

The ER is the largest organelle in eukaryotic cells. The peripheral ER is composed of a single continuous membrane that branches from the nuclear envelope and consists of two main structural domains, flat membrane cisternae (also called sheets) and tubules, which are dynamic structures (6). ER sheets are covered with ribosomes and support synthesis, translocation, and folding of proteins. Tubules, whose function is less well understood, associate with significantly fewer ribosomes and may be sites for lipid synthesis and communication with other organelles (7). It is not known how the ER maintains this dynamic network of sheets and tubules, although the process requires contributions from motor proteins, the cytoskeleton, proteins that mediate ER-ER fusion, and membrane-bending proteins (8). Collectively, ER shape is influenced by a surprisingly small set of proteins (9).

Viruses often interfere with the dynamic organization of the ER. Viruses use ER membranes and ribosomes to protect the viral genome and synthesize viral proteins (10, 11). Viruses also remodel ER membranes to form a variety of structures, including single-membrane spherule vesicles in the ER lumen (12, 13), double-membrane vesicles (DMVs) (14, 15), convoluted membranes (CMs) (16), and single-membrane sheets (17). Spherules and DMVs appear to function in viral genome replication, CMs are likely sites of polyprotein synthesis or storage of proteins and lipids, and single-membrane sheets may participate in viral morphogenesis. How viral proteins remodel the ER during viral infection is largely unknown.

Mammalian reoviruses are nonenveloped, double-stranded RNA (dsRNA) viruses that replicate in a wide range of cells and tissues (18). These viruses infect humans throughout their lifetime (19) and have been implicated in the pathogenesis of celiac disease (20). Reovirus replication, transcription, and assembly occur in large cytoplasmic structures termed viral inclusions (VIs) (21). Inclusions were generally thought to be membrane-free structures, but they contain smooth membranes attached to mitochondria (22). ER cisternae surround reovirus inclusions, and ribosomes are distributed within these structures (21, 22). These findings point to the potential participation of the ER in VI formation and architecture.

In this study, we discovered that major remodeling of the ER during reovirus infection leads to collections of vesicles and tubules that form the inclusion structures. These membranous components remain attached to the remodeled ER to build large replication factories. Remarkably, expression of reovirus proteins σNS and μNS is sufficient to mediate this dramatic reorganization of the ER. Moreover, we demonstrate that σNS causes ER tubulation and μNS causes ER fragmentation. Our results enhance an understanding of how the ER is reshaped and transformed by viruses and point to a new process used by viruses to form factories for particle assembly.

## RESULTS

### Remodeled ER membranes associate with reovirus inclusions.

**FIG 1:**
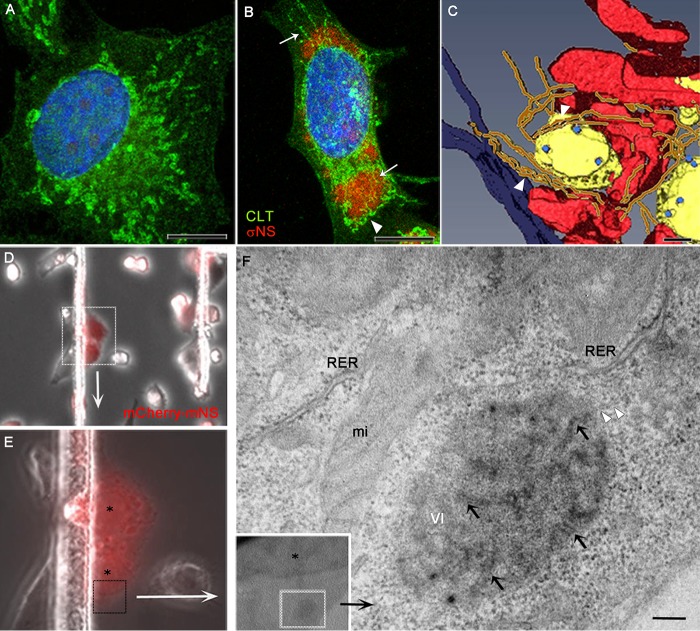
ER remodeling in reovirus-infected cells as visualized by confocal microscopy, 3D TEM, and CLEM. HeLa cells were adsorbed with reovirus T1L M1-P208S. At 14 h postadsorption, cells were immunolabeled with a rabbit anti-calreticulin (CLT) polyclonal antiserum, a mouse anti-σNS monoclonal antibody, and the corresponding secondary antibodies conjugated with Alexa 488 (green) and Alexa 594 (red). Nuclei were stained with DAPI (blue). (A) A mock-infected cell with normal ER cisternae. (B) Reovirus-infected cell with altered ER. White arrows indicate thin, fragmented ER membranes around and inside VIs. The arrowhead indicates thin, undulated ER attached to a VI. (C) TEM of serial sections and 3D reconstruction. VIs (yellow) containing viral particles (light blue) are surrounded by a network of abnormally thin, undulated ER cisternae (brown) that contact the VI (arrowheads). Mitochondria are colored in red, and the nuclear envelope is in dark blue. (D to F) CLEM of reovirus inclusions. HeLa cells engineered to express mCherry-μNS-MT were adsorbed with reovirus, incubated for 14 h, and imaged using bright-field and fluorescence microscopy. Cell nuclei are labeled with asterisks. Selected fluorescent cells (dashed squares) were imaged using TEM (F). An early VI is surrounded by rough ER (RER) and mitochondria (mi). Membranes distribute inside (black arrows) and at the periphery (arrowheads) of the inclusion. Bars, 10 µm (A and B), 500 nm (C), and 200 nm (F).

ER cisternae surround and contact reovirus inclusions in HeLa and MDCK cells (22). Here, we studied the association of ER with VIs by confocal and electron microcopy (Fig. 1). The ER was immunolabeled with an antibody specific for the ER membrane marker calreticulin. Fluorescence confocal microscopy showed that reovirus infection triggers a massive transformation of the ER that becomes thin, undulated, and fragmented (Fig. 1A and B). Three-dimensional (3D) reconstructions of serial transmission electron microscopy (TEM) images showed that groups of thin, undulated ER tubules concentrate around VIs (Fig. 1C). Similar findings were obtained in studies of reovirus-infected mouse embryo fibroblasts (MEFs) (see Fig. S1 in the supplemental material). ER membranes were observed surrounding and inside VIs using confocal microscopy (Fig. S1A). TEM of VIs at various magnifications showed rough ER (RER) surrounding and contacting the VIs, and membrane fragments were often seen inside VIs (Fig. S1B).

10.1128/mBio.01253-18.1 FIG S1  Reovirus inclusions in MEF cells. Cells were adsorbed with reovirus T1L M1-P208S at an MOI of 1 PFU/cell, incubated for 14 h, and imaged using fluorescence microscopy (A) and TEM (B). (A) Immunofluorescence imaging using a rabbit polyclonal anticalreticulin (CLT) antiserum and a secondary antibody conjugated with Alexa 488 (green) and a mouse monoclonal anti-σNS antibody followed by a secondary antibody conjugated with Alexa 594 (red) shows ER labeling in reovirus inclusions (image on the left, arrows). The enlarged image on the right shows ER labeling around and inside the VI (arrows). (B) TEM of viral inclusions (VIs) at various magnifications showing RER surrounding and contacting the VIs. There are abundant membranes inside the VIs (arrows), viral particles (black arrowheads), and ribosomes at the periphery (white arrowheads). Bars, 10 µm (A), 100 nm (B). Download FIG S1, PDF file, 2.4 MB. Copyright © 2018 Tenorio et al. 2018 Tenorio et al. This content is distributed under the terms of the Creative Commons Attribution 4.0 International license.

To determine whether remodeled ER membranes associate with reovirus inclusions early in infection, we used correlative light and electron microscopy (CLEM). HeLa cells expressing mCherry fused to the first 230 amino acids of μNS, which incorporate a domain required to interact with several other viral proteins and localize to VIs (23), were cultured on photoetched gridded coverslips, infected with reovirus, and imaged using fluorescence microscopy (Fig. 1D and E). Fluorescent mCherry-μNS protein was recruited to nascent VIs that were selected for ultrastructural analysis. TEM of ultrathin sections showed membranes inside and at the periphery of early VIs (Fig. 1F).

### ER proteins and newly synthesized viral RNA are detected inside VIs.

**FIG 2:**
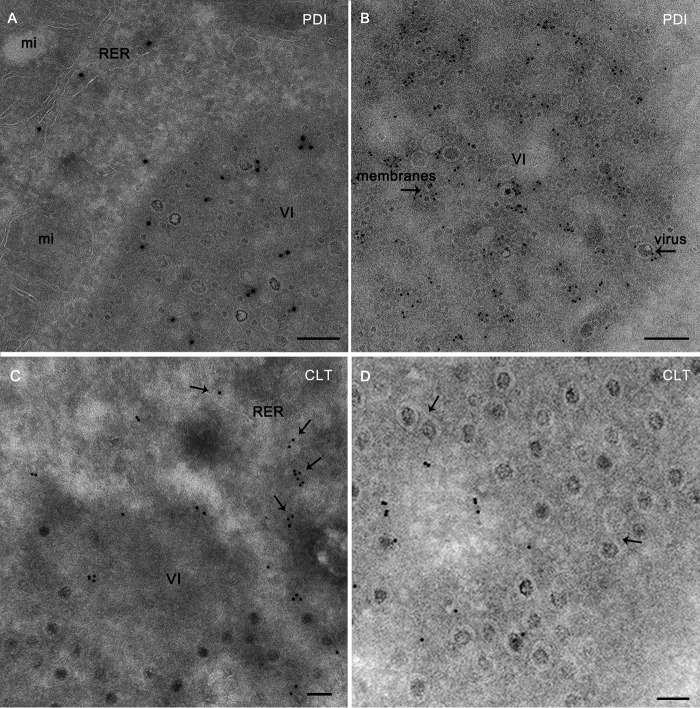

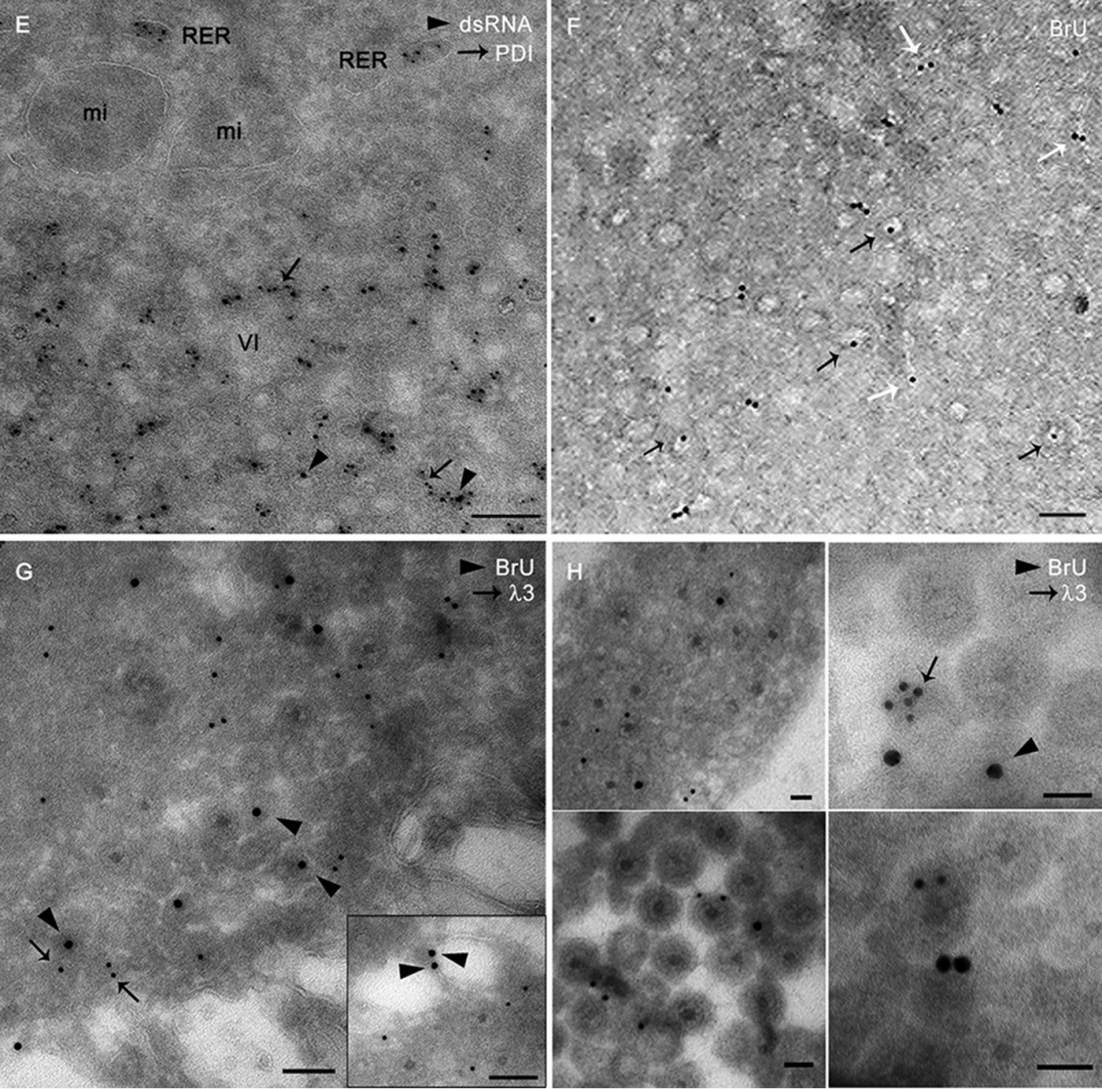
Immunogold labeling of ER proteins in Tokuyasu cryosections of reovirus inclusions. HeLa cells were adsorbed with reovirus and incubated for 24 (A, B, and E) or 14 (C, D, and F to H) h, frozen in liquid nitrogen, and sectioned at −120°C. (A to D) Thawed cryosections were processed for immunogold labeling using a rabbit polyclonal anti-PDI antiserum and a secondary antibody bound to 10-nm colloidal gold particles (A and B) or a rabbit polyclonal anti-calreticulin (CLT) antiserum and a secondary antibody bound to 10-nm colloidal gold particles (C and D). (E) Cryosections were double labeled with a rabbit polyclonal anti-PDI antiserum and a secondary antibody bound to 5-nm colloidal gold particles (arrows) and a mouse monoclonal anti-dsRNA antibody and a secondary antibody bound to 15-nm colloidal gold particles (arrowheads). Anti-PDI antibody labels RER cisternae in the cytosol and membranes inside inclusions. Anti-dsRNA antibody labels membranes and viral particles inside VIs. (F) Cryosection labeled with a mouse monoclonal anti-BrU antibody and a secondary antibody bound to 10-nm colloidal gold particles. The anti-BrU antibody labels membrane fragments (white arrows) and viral particles (black arrows). (G and H) Cryosections were double labeled with a mouse monoclonal anti-BrU antibody and a secondary antibody bound to 15-nm colloidal gold particles (arrowheads) and an antiserum specific for the λ3 viral RNA-dependent RNA polymerase and a secondary antibody bound to 5-nm colloidal gold particles (arrows). Low- and high-magnification views of VIs show that both antibodies label viral particles and membrane fragments (inset in panel G). Bars, 200 nm (A, B, and E), 100 nm (C, D, F, G, and inset in G), and 50 nm (H).

The Tokuyasu cryosectioning technique does not require sample dehydration and provides optimal preservation of membranes and the highest accessibility of antigens to antibodies for immunostaining (24, 25). We prepared Tokuyasu cryosections of reovirus-infected cells for immunogold labeling of protein disulfide isomerase (PDI) and calreticulin, luminal, and membrane ER proteins, respectively (Fig. 2). Both anti-PDI (Fig. 2A and B) and anti-calreticulin (Fig. 2C and D) antibodies labeled characteristic ER cisternae in the cytosol as well as membrane fragments, vesicles, and viral particles inside inclusions.

To determine whether tubulovesicular elements of the ER-Golgi intermediate compartment (ERGIC) are present inside VIs, we immunogold labeled the ERGIC marker KDEL-R. In mock-infected and reovirus-infected cells, KDEL-R was detected in pre-Golgi and *cis*-Golgi membranous elements but not in VIs (data not shown).

Immunogold labeling with an antibody specific for dsRNA that labels viral replication sites (26, 27) and reovirus ribonucleoproteins (vRNPs) showed signals in viral particles and membranes inside the VIs (Fig. 2E). Colocalization of dsRNA with PDI also was observed (Fig. 2E). To localize viral RNA synthesis relative to VIs, we assessed bromouridine (BrU) incorporation via immunogold labeling of Tokuyasu cryosections (Fig. 2F). BrU labeling was observed in inclusions associated with viral particles, marking viral RNA that had accumulated during the 5-h labeling window (Fig. 2F). These particles are viral cores in which genome replication takes place (28, 29) but also likely mature virions, which may retain signal due to the prolonged labeling window. BrU labeling also was associated with membranes inside VIs (Fig. 2F). Although BrU was maintained in the cell culture medium for 5 h, BrU signal was found only in VIs. A total of 258 labeled structures in VIs from 5 cells were photographed. BrU labeling was detected in viral particles (35%) and membranes (46%) as well as in some labeled structures that were not clearly identified (19%). Double labeling with antibodies specific for BrU and the λ3 viral RNA-dependent RNA polymerase, which resides in the virion core, showed that inside VIs, both antibodies label viral particles (Fig. 2G and H) and membrane fragments (Fig. 2G, inset). These results suggest that the ER-derived, tubulovesicular membranes inside VIs support viral replication and assembly. Considering that genome replication of *Reoviridae* viruses occurs inside viral cores (28, 29), the significance of membrane fragments labeled with anti-dsRNA or anti-BrU antibodies inside VIs is uncertain. Nonetheless, our findings raise the possibility that reovirus genome segments associate with membranes prior to assortment into viral particles inside VIs.

**FIG 3:**
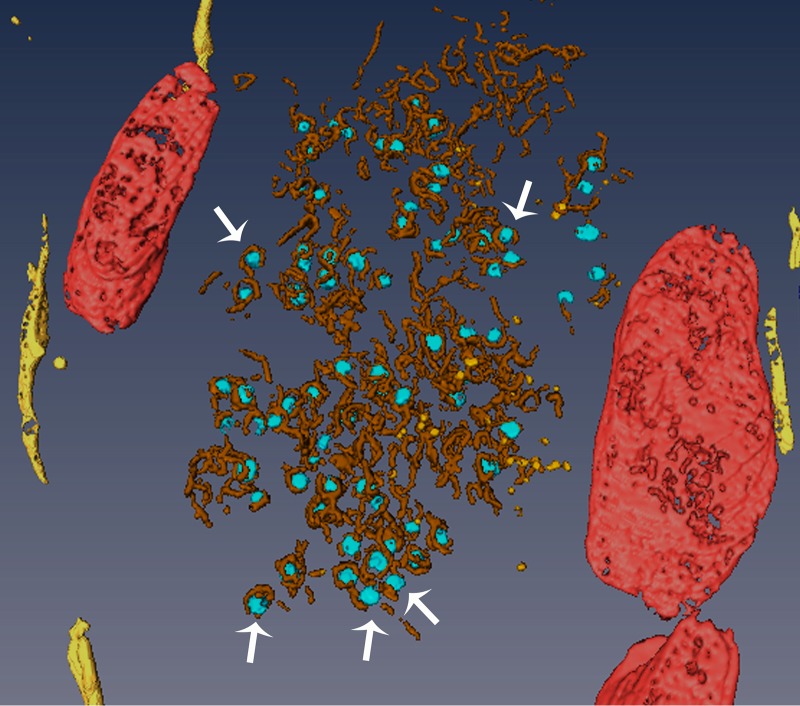
Electron tomography of a reovirus inclusion. HeLa cells were adsorbed with reovirus, incubated for 14 h, frozen in liquid nitrogen, and sectioned at −120°C. Thawed cryosections were processed by electron tomography. A single-axis tilt series was obtained between −63° and +60° with an angular interval of 1.5°. Images were recorded using an Eagle 4k-by-4k slow-scan charge-coupled device (FEI, Eindhoven, The Netherlands) with FEI software and a Tecnai G2 microscope (FEI) operating at 200 kV. Images were aligned, and the tomogram was reconstructed using the IMOD software package. The tomogram was subjected to noise filtering and automated segmentation to visualize membranes. The 3D model was constructed using Amira. RER, yellow; viral particles, light blue; mitochondria, red; tubules and membrane fragments inside the VI, brown; vesicles inside the VI, orange. The VI is a collection of vesicles and tubules with viral particles attached to membranes (arrows).

The 3D membranous internal organization of VIs was analyzed in detail by electron tomography of Tokuyasu cryosections. The tomographic volumes revealed that VIs consist of groups of thin tubules and vesicles (Fig. 3). The diameter of the thin tubules is approximately one-third of the normal ER cisternae. Mitochondria and ER cisternae are adjacent to the inclusions. Remarkably, all viral particles inside the VIs are attached to membranes (Movies S1 and S2). We conclude that the membranous compartment primary involved in construction of reovirus inclusions is the ER.

10.1128/mBio.01253-18.6 MOVIE S1  3D model of a reovirus inclusion visualized by electron tomography (related to Fig. 3). The 3D model was constructed using e-tomo and a combination of masking, isosurface, and manual tracing with Amira segmentation tools. Black spots represent gold particles used as fiducials. The computational slices of the tomogram are first swept upwards (first third of the movie) and then backwards (second third), revealing the 3D isosurface representation. The last third of the movie rotates the 3D representation. ER, light yellow; viral particles, light blue; nuclear membrane, dark blue; mitochondria, red; membrane fragments, brown; vesicles, orange. Download MOVIE S1, AVI file, 16.2 MB. Copyright © 2018 Tenorio et al. 2018 Tenorio et al. This content is distributed under the terms of the Creative Commons Attribution 4.0 International license.

10.1128/mBio.01253-18.7 MOVIE S2  Detail of the membrane network segmentation in a reovirus inclusion. A computational tomographic slice and the corresponding 3D model (from Movie S1) are shown. Note that fiducials (black spots) do not interfere with membrane segmentation. Download MOVIE S2, MPG file, 3.2 MB. Copyright © 2018 Tenorio et al. 2018 Tenorio et al. This content is distributed under the terms of the Creative Commons Attribution 4.0 International license.

### ER remodeling during the initial stages of reovirus infection.

**FIG 4:**
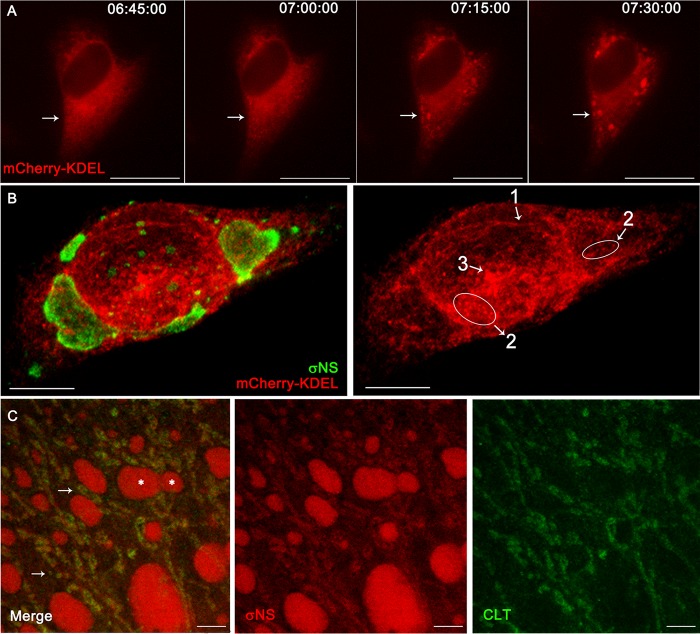
Live-cell microscopy, confocal microscopy, and STED of ER remodeling during reovirus infection. (A) HeLa cells were transfected with mCherry-KDEL, adsorbed with reovirus, and incubated for 24 h. Images were collected every 15 min. A cell is shown at 6 h 45 min, 7 h, 7 h 15 min, and 7 h 30 min postadsorption. Normal ER elements are progressively fragmented, collapsed, and aggregated (arrows). (B) HeLa cells were transfected with mCherry-KDEL and, at 24 h posttransfection, adsorbed with reovirus, incubated for 24 h, and imaged using confocal microscopy. The ER in infected cells is progressively thinned (1), fragmented (2), and collapsed and aggregated (3). (C) STED microscopy of reovirus-infected cells. HeLa cells were adsorbed with reovirus and fixed at 26 h. Cells were immunolabeled with σNS-specific antibody and calreticulin (CLT)-specific antibody followed by secondary antibodies conjugated with Alexa 488 (green) and Alexa 546 (red). σNS (red) associates with remodeled ER (green). Some VIs are marked with white asterisks. σNS and ER marker CLT colocalize (arrows) over the network of remodeled ER with VIs. Bars, 10 µm (A), 7.5 µm (B), and 2.5 µm (C).

To visualize ER remodeling during reovirus infection, HeLa cells were transfected with mCherry-KDEL and adsorbed with reovirus. ER remodeling during infection was visualized using real-time, live-cell microscopy. Early in infection, the ER fragmented, collapsed, and aggregated (Fig. 4A; Movie S3). Immunofluorescence and confocal microscopy imaging after video recording confirmed that cells with the observed ER remodeling were indeed infected (Fig. S2; Movie S4).

10.1128/mBio.01253-18.2 FIG S2  Immunofluorescence and confocal microscopy of infected cells after video recording (related to Movie S4). HeLa cells shown in Movie S4 were stained with a σNS-specific antibody and a secondary antibody conjugated with Alexa 488 (green). (A) Three individual frames from Movie S4 are shown. Arrows point to a cell with remodeled ER. (B) Last frame of the movie (left) and confocal micrographs (center and right) of the same cell (arrows) following immunofluorescence staining. Bars, 10 µm. Download FIG S2, PDF file, 1.9 MB. Copyright © 2018 Tenorio et al. 2018 Tenorio et al. This content is distributed under the terms of the Creative Commons Attribution 4.0 International license.

10.1128/mBio.01253-18.8 MOVIE S3  Video showing ER remodeling in a reovirus-infected HeLa cell (related to Fig. 4). Cells were transfected with mCherry-KDEL and adsorbed with reovirus T1L M1-P208S at an MOI of 1 PFU/cell. Images were collected every 15 min from 5 to 10 h postadsorption and processed using LAS X software. Download MOVIE S3, AVI file, 9.2 MB. Copyright © 2018 Tenorio et al. 2018 Tenorio et al. This content is distributed under the terms of the Creative Commons Attribution 4.0 International license.

10.1128/mBio.01253-18.9 MOVIE S4  Video showing ER remodeling in a reovirus-infected HeLa cell (related to Fig. S2). Cells were transfected with mCherry-KDEL and adsorbed with reovirus T1L M1-P208S at an MOI of 1 PFU/cell. Images were collected every 15 min from 5 to 10 h postadsorption and processed using LAS X software. Download MOVIE S4, AVI file, 3.8 MB. Copyright © 2018 Tenorio et al. 2018 Tenorio et al. This content is distributed under the terms of the Creative Commons Attribution 4.0 International license.

For a more precise, higher-resolution analysis of ER remodeling during infection, we used confocal microscopy to image HeLa cells transfected with mCherry-KDEL and infected with reovirus (Fig. 4B). ER remodeling in infected cells was found to occur by a process that begins with tubule thinning, followed by fragmentation, and concludes with collapse. Similar findings were obtained using live-cell imaging and confocal microscopy of U-2 OS cells engineered to stably express mCherry-KDEL, transfected with an N-terminal-tagged green fluorescent protein (GFP) construct expressing residues 1 to 230 of the µNS protein, and infected with reovirus (Movie S5). Live-cell imaging showed that VIs interact with the remodeled ER during infection.

10.1128/mBio.01253-18.10 MOVIE S5  Live-cell imaging of reovirus infection demonstrates altered ER morphology in U-2 OS cells. U-2 OS cells engineered to stably express mCherry-KDEL were transfected with an N-terminally tagged GFP construct expressing residues 1 to 230 of the µNS protein. Cells were adsorbed with reovirus T1L at an MOI of 10,000 PFU/cell and imaged every 30 min from 9 to 18 h postinfection. VIs (in green) interact with the remodeled ER (in red) during infection. Download MOVIE S5, AVI file, 1 MB. Copyright © 2018 Tenorio et al. 2018 Tenorio et al. This content is distributed under the terms of the Creative Commons Attribution 4.0 International license.

Stimulated emission depletion (STED) superresolution microscopy revealed additional fine details of the remodeled ER in reovirus-infected cells (Fig. 4C). The reovirus σNS protein was found to associate with ER thin tubules and fragments surrounding and inside nascent, small inclusions (arrows in Fig. 4C). VIs of a variety of sizes remained attached to the remodeled ER. Based on these observations, we conclude that the ER undergoes rapid remodeling in an ordered process after infection and that VIs associate with the remodeled ER to build large inclusions and replication factories.

### Reovirus σNS and μNS remodel the ER.

**FIG 5:**
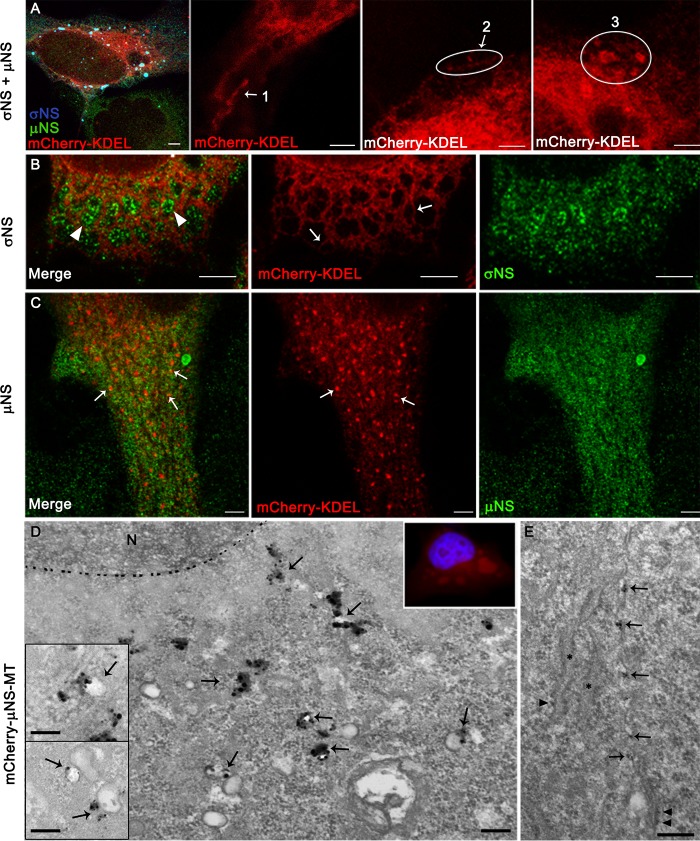
Effect of T3D σNS and μNS expression on ER morphology. (A) HeLa cells were transfected with mCherry-KDEL, σNS, and μNS and, at 24 h posttransfection, imaged using immunofluorescence and confocal microscopy. ER alterations are similar following cotransfection of σNS and μNS and reovirus infection: (1) linear thinning, (2) fragmentation, and (3) collapse and aggregation. (B and C) HeLa cells were transfected with mCherry-KDEL in combination with σNS (B) or μNS (C) and imaged using confocal microscopy at 24 h posttransfection. (B) Cells expressing σNS showed an altered ER with long, separated, branched thin tubules (arrows). σNS concentrates in the gaps between the tubules, producing a ring-like pattern (arrowheads). (C) Cells expressing μNS showed an altered ER with μNS associated with ER fragments (arrows). (D) HeLa cells engineered to express mCherry-μNS-MT were adsorbed with reovirus, incubated for 8 h, fixed, incubated with gold and silver, and imaged using fluorescence microscopy and TEM. mCherry-μNS-MT gold-silver distributes with vesicles (arrows) near the nucleus (N) where the Cherry red fluorescent signal concentrates (inset on the right; nucleus in blue). Insets on the left show vesicles with mCherry-μNS-MT gold-silver from different cells. The dashed line marks the periphery of the nucleus. (E) mCherry-μNS-MT gold-silver also localizes to strangled ER cisternae (arrows). Asterisks mark normal ER cisternae, and arrowheads indicate ribosomes adjacent to normal or thin, strangled ER cisternae. Bars, 5 µm (A), 3 µm (B), 2.5 µm (C), and 200 nm (D and E and insets in D).

To identify the viral proteins that induce ER remodeling, we ectopically expressed the reovirus σNS and µNS proteins and monitored ER morphology. Both proteins distribute to reovirus inclusions (30, 31), although precise functions for each are not well understood. HeLa cells were transfected with mCherry-KDEL, σNS, and μNS and imaged by confocal microscopy. Experiments with expression plasmids encoding σNS and μNS from reovirus strains T1L and T3D produced similar results (Fig. 5 and S3). The expression of these viral proteins caused ER remodeling like that observed during reovirus infection, with ER tubulation followed by fragmentation and culminating in collapse (Fig. 5A).

10.1128/mBio.01253-18.3 FIG S3  Effect of T1L and T3D σNS and µNS expression in ER morphology. HeLa cells were transfected with mCherry-KDEL (A and E) or mCherry-KDEL in combination with T1L σNS (B), T3D σNS (C and D), T1L μNS (F and G), or T3D μNS (H and I) and imaged using confocal microscopy at 24 h posttransfection. Cells expressing σNS show an altered ER with long, separated, branched thin tubules (white ellipses in panel B, arrows in panel D). μNS associates with thin ER tubules (arrows in panels G and H) and fragments (arrows in panels F and I). Bars, 10 µm (A and E), 8 µm (B and C), and 2.5 µm (F to I). Download FIG S3, PDF file, 2 MB. Copyright © 2018 Tenorio et al. 2018 Tenorio et al. This content is distributed under the terms of the Creative Commons Attribution 4.0 International license.

To determine the effect of independent expression of σNS and μNS on alterations in ER structure, HeLa cells were transfected with mCherry-KDEL alone or in combination with either σNS or μNS and imaged by confocal microscopy 24 h posttransfection. Cells expressing σNS showed an altered ER with separated, thin, branched tubules (Fig. 5B and S3). The σNS protein concentrated in the gaps between the tubules, producing a ring-like pattern (Fig. 5B). Cells expressing μNS showed long, thin ER tubules without branches (Fig. S3) and a fragmented ER (Fig. 5C). μNS associated with ER tubules and fragments (Fig. 5C and S3).

**FIG 6:**
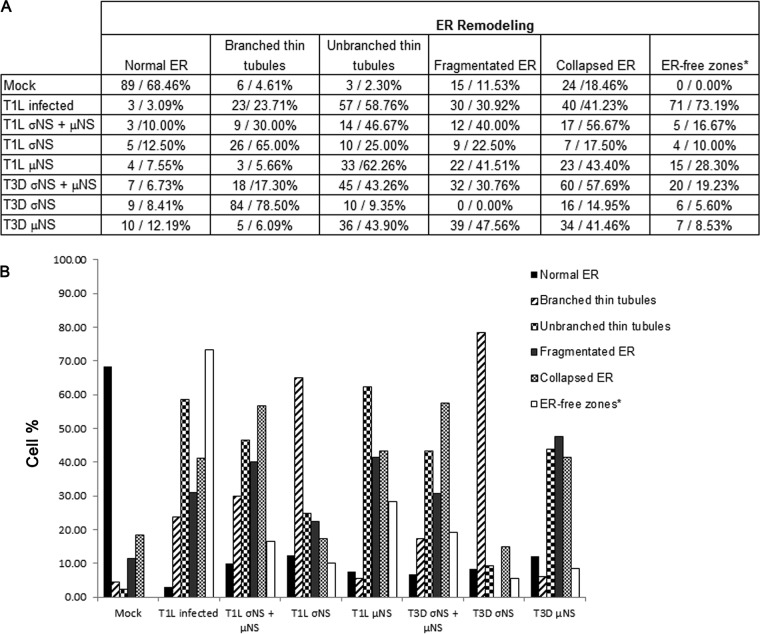
Quantitative confocal microscopy data. (A) Summary showing the number and percentage of cells with various ER morphologies and alterations (normal ER, branched thin tubules, unbranched thin tubules, fragmented ER, and collapsed ER) under different experimental conditions: mock infected, virus infected, cotransfected with σNS and μNS, transfected with σNS alone, and transfected with μNS alone. Large ER-free zones (*) are areas of the cell with a surface of ≥15 µm^2^ that contain few or no ER elements, like those in Fig. 5A. (B) Comparative analysis of ER alterations under different experimental conditions. Cells at 14 h postinfection and 24 h posttransfection were included in the quantification. This quantitative analysis confirms that σNS causes a thinning of the tubular ER, whereas μNS disrupts the branches of the thin tubules and fragments those structures into small pieces. At later stages of infection, the ER collapses and aggregates, leaving large gaps in the cytosol.

To investigate how μNS induces ER fragmentation, we localized μNS molecules with metal-tagging TEM (METTEM), a highly sensitive labeling technique (32, 33). HeLa cells engineered to express mCherry fused to μNS residues 1 to 230 and metallothionein (MT) (mCherry-μNS-MT) were adsorbed with reovirus, incubated with gold and silver, fixed, and embedded in resin. Ultrathin sections were stained and imaged using TEM (Fig. 5D and S4). Gold-silver particles bound to MT revealed the precise location of MT-tagged μNS molecules. We observed that prior to VI assembly, mCherry-μNS-MT distributes with vesicles near the nucleus where the mCherry fluorescent signal concentrates (Fig. 5D); mCherry-μNS-MT gold-silver molecules also distributed to thin, strangled ER cisternae where they distribute with near-uniform spacing (Fig. 5E). Together with the confocal images shown in Fig. 5C, this finding suggests a role for μNS in ER fragmentation. Results from gene silencing experiments using either σNS- or μNS-specific small interfering RNAs (siRNAs) in reovirus-infected cells showed only minor ER remodeling. This finding is consistent with the observation that μNS expression is diminished following σNS silencing and vice versa (Fig. S5). The ER morphological changes caused by reovirus infection or ectopic expression of σNS and μNS were quantified microscopically (Fig. 6). In this analysis, σNS causes a thinning of the tubular ER, whereas μNS disrupts the branches of the thin tubules and cleaves those structures into small pieces. At later stages of infection, the ER collapses and aggregates, leaving large gaps in the cytosol.

10.1128/mBio.01253-18.4 FIG S4  TEM detection of mCherry-μNS-MT gold-silver is specific. (A) Perinuclear area of mock-transfected, mock-infected HeLa cells incubated with gold and silver before embedding, thin sectioning, and imaging using TEM. The nucleus (N) and all cytosolic structures are free of label. (B) Perinuclear region of a HeLa cell engineered to express mCherry-μNS-MT, adsorbed with reovirus, and incubated with gold and silver before embedding, sectioning, and imaging. Signals reveal the distribution of mCherry-μNS-MT gold-silver in vesicles of variable size (arrows). mi, mitochondria. Bars, 200 nm. Download FIG S4, PDF file, 0.9 MB. Copyright © 2018 Tenorio et al. 2018 Tenorio et al. This content is distributed under the terms of the Creative Commons Attribution 4.0 International license.

10.1128/mBio.01253-18.5 FIG S5  Effect of reovirus σNS and μNS silencing on ER morphology in infected HeLa cells. (A) HeLa cells transfected with a luciferase-specific siRNA display a normal ER organization. (B) Immunoblot following σNS silencing (a) and μNS silencing (b). Lanes 1 and 2, mock-infected cells; lane 3, infected cells; lane 4, infected and silenced cells. The immunoblot shows that μNS expression is diminished following σNS silencing and vice versa. (C) Cells adsorbed with reovirus T1L M1-P208S at an MOI of 1 PFU/cell and incubated for 24 h form characteristic VIs and display a significantly altered ER (dashed ellipse). (D) Cells transfected with σNS-specific siRNA, adsorbed with reovirus T1L M1-P208S at an MOI of 1 PFU/cell, and incubated for 24 h form small VIs. The ER has some alterations, including the formation of thin tubules and collapse (arrows). (E) Cells transfected with μNS-specific siRNA, adsorbed with reovirus T1L M1-P208S at an MOI of 1 PFU/cell, and incubated for 24 h do not produce VIs. The ER is altered at the cell periphery with separated thin tubules (dashed ellipses). Bars, 10 µm. Download FIG S5, PDF file, 2.6 MB. Copyright © 2018 Tenorio et al. 2018 Tenorio et al. This content is distributed under the terms of the Creative Commons Attribution 4.0 International license.

## DISCUSSION

**FIG 7:**
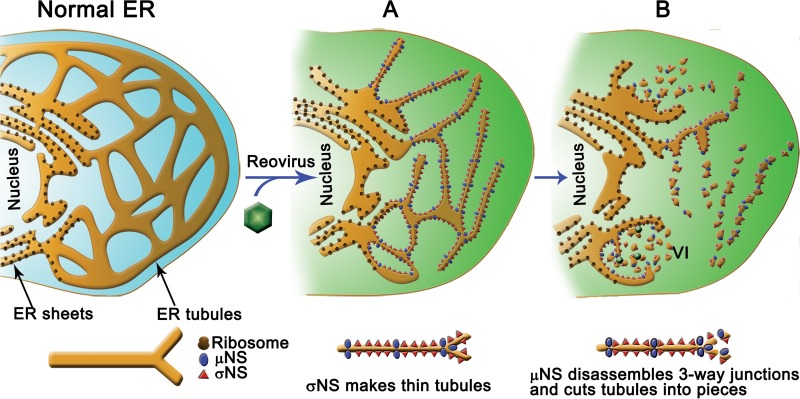
Model of ER remodeling induced by reovirus infection and the specific action of σNS and μNS. Normal ER is composed of ER sheets and tubules. Reovirus targets ER tubules, leaving the sheets untouched. (A) Early in infection, σNS binds to ER cisternae and transforms these structures into thin tubules. (B) μNS binds to thin tubules, eliminates their branches, and severs them into small membranous pieces that aggregate, attach to the remodeled ER, and form VIs. Inside inclusions, replicating viral cores and newly synthesized vRNPs bind to membranes that most likely serve as assembly sites for new viral particles. Schematics at the bottom show how σNS and μNS might remodel ER tubules.

In this study, we demonstrate that the membranes in reovirus inclusions (22) originate by ER tubulation and fragmentation, and we provide evidence that the reovirus σNS and μNS proteins are responsible for this remodeling. We also discovered that the collections of ER-derived membranous elements that form inclusions are not free in the cytosol but remain associated with a net of remodeled ER tubules that forms the internal organization of the inclusion structure. This VI/ER association most likely facilitates the incorporation of newly synthesized viral proteins and RNAs into nascent particles (21). A model of VI formation consistent with our findings is shown in Fig. 7.

The peculiar and massive ER remodeling induced by reovirus has not been reported for any virus. Reovirus VIs have been mainly studied using fluorescence microscopy and TEM of ultrathin sections. It was concluded from these studies that reovirus VIs are isolated structures unmoored in the cytoplasm. We employed a more comprehensive imaging approach to investigate reovirus inclusions that included real-time, live-cell imaging, confocal and STED superresolution microscopy, CLEM, 3D EM and electron tomography, quantitative microscopy, and two highly sensitive methods for molecular mapping *in situ*. Results gathered using these complementary techniques have provided us with a new understanding of the organization and biogenesis of reovirus inclusions.

Other members of the *Reoviridae* use membranes during their life cycle. In polarized intestinal Caco-2 cells, rotavirus particles enter ER cisternae on the periphery of rotavirus factories, which are called viroplasms. Virions later exit the ER and incorporate into small smooth vesicles that are transported to the cell surface. This nonconventional vesicular transport mechanism bypasses the Golgi complex and mediates rotavirus nonlytic egress (34). Rotavirus RNA synthesis occurs in viroplasms (35), which are dynamic structures that move perinuclearly during infection and fuse with each other in a process dependent on microtubules (36, 37). To generate a unifying model of *Reoviridae* inclusion morphogenesis, it will be important to determine whether viroplasms formed during rotavirus infection (as well as the factories formed by other members of the *Reoviridae*) also consist of membranes.

Although many viruses partition the ER to form replication organelles, we do not know how ER remodeling is induced in infected cells. In this regard, only a few cellular proteins are known to participate in ER transformation during viral infection. Reticulons, which are ER-shaping proteins, function in the assembly and stabilization of spherules containing viral replication complexes of brome mosaic virus (38). Reticulons also induce tubules and vesicles of positive curvature and enhance replication of enteroviruses (39). Rab18, a small GTPase that cycles between the cytosol, ER, and lipid droplets, participates in the recruitment of lipid droplets to hepatitis C virus replication sites in ER membranes, bringing together several components required for viral replication and morphogenesis (40). Finally, the ER-resident vesicle-associated membrane protein (VAMP)-associated protein and oxysterol-binding protein, a lipid transfer protein located at ER-Golgi membrane contact sites, are used by several RNA viruses to mediate lipid exchange between the ER and other organelles (41, 42). These lipid flows modify membrane composition and stabilize viral replication complexes (43). However, viral proteins that induce ER remodeling are not known.

Our findings provide clues about the functions of two poorly understood reovirus replication proteins. Reovirus nonstructural proteins σNS and μNS are the minimal viral components required to assemble inclusions. Although the precise functions of these proteins are not clear, each is required for viral genome replication (44). Our study demonstrates that the ER remodeling necessary to build reovirus inclusions is mediated by these proteins. By expressing these proteins together or individually in the absence of viral infection, we found that σNS transforms the ER into thin tubules, while μNS eliminates branches and fragments the tubules into small pieces. Both proteins likely modify the ER simultaneously during infection, with σNS inducing tubulation and μNS disturbing the three-way junctions to produce unbranched, thin tubules, followed by scission of the tubules into smaller fragments (Fig. 7). Thinning of the ER by σNS may generate tension in the tubules just before fragmentation. Since μNS also can mediate formation of thin tubules, the synergistic effect of both proteins in thinning the ER likely facilitates tubule fragmentation by μNS. σNS and μNS could transform the ER by interacting with lipids in ER membranes or interfering with ER-shaping proteins, such as reticulons, Rab GTPases, or lunapark (8). μNS also could target proteins at the three-way junctions such as atlastins (45). Although confocal and superresolution STED microscopy showed that during infection σNS and μNS distribute to the ER in abundance, the protein concentration required for the observed ER remodeling need not be large. For example, occupation of as little as 10% of the tubular ER surface by bending proteins can induce pronounced ER curvature in yeast (46).

Contrary to the currently accepted concept of reovirus inclusions as isolated neo-organelles assembled in the cytoplasm of infected cells, we found that reovirus replication factories are comprised of remodeled peripheral ER with attached VIs as active domains formed by clusters of ER-derived vesicles and tubules. Our work uncovers a new mechanism by which viruses form neo-organelles for particle assembly and a new type of virus-induced ER remodeling. The large membrane surface generated by ER fragmentation would provide an adequate shelter for reovirus replication complexes, which are protected inside viral cores (18, 47), as well as the reovirus translation machinery (21). We hypothesize that (i) viral cores actively replicating the genome are bound to membranous tubules, (ii) newly synthesized viral RNAs exit the cores and attach to these membranes via specific interactions with viral proteins, and (iii) assembly of the inner core and outer capsid to build progeny viral particles occurs around vRNPs anchored to these sites (Fig. 7). High-resolution studies showing the precise localization and movement of molecules inside VIs will be required to understand how viral genome replication and particle assembly are coupled inside the VIs.

## MATERIALS AND METHODS

### Cells, viruses, and plasmids.

HeLa CCL2 cells were grown in Dulbecco’s modified Eagle’s medium (DMEM; D6429; Sigma) supplemented to contain 10% fetal bovine serum, 100 U/ml penicillin G, 100 µg/ml streptomycin (Gibco), 0.25 µg/ml amphotericin B, nonessential amino acids, 2 mM l-glutamine, and 1 mM sodium pyruvate (Sigma).

Engineered mCherry-T1LM3 230MT-HeLa CCL2 cells stably expressing viral μNS protein fused to mCherry and a metallothionein (MT) tag were generated by transducing cells with replication-incompetent retrovirus. Cells were then cultured in the same medium supplemented with 1 µg/ml puromycin (Sigma). L929 cells and MEFs were grown in Dulbecco’s modified Eagle’s medium (D6429; Sigma) supplemented to contain 10% fetal bovine serum, 100 U/ml penicillin G, 100 µg/ml streptomycin (Gibco), nonessential amino acids (Sigma), and 2 mM l-glutamine.

U-2 OS cells stably expressing mCherry-KDEL (U-2 OS mCherry-KDEL cells) were provided by Carolyn Coyne (University of Pittsburgh). U-2 OS mCherry-KDEL cells were grown in Dulbecco’s modified Eagle’s medium supplemented to contain 5% fetal bovine serum, 100 U/ml penicillin G, 100 µg/ml streptomycin (Gibco), 0.25 µg/ml amphotericin B, and 50 µg/ml of G418 sulfate (Thermo Fisher).

Cells were infected with reovirus strain T1L M1-P208S, which is identical to the prototype T1L strain except for a proline-to-serine mutation at position 208 of the µ2 protein (M1 gene). This mutation changes inclusion morphology from filamentous to globular (48). This virus was recovered using reverse genetics (49). Site-directed mutagenesis was used to engineer the P208S substitution in the M1 gene with the following primers: forward, 5′ CATTTCGGGGTAGCAATTGATGAAAATGTGCCAACATTAAATCTAG 3′; reverse, 5′ CTAGATTTAATGTTGGCACATTTTCATCAATTGCTACCCCGAAATG. Virus was purified by cesium gradient centrifugation (50). Viral titers were determined by plaque assay using L929 cells (51).

Reovirus T1L σNS (52) and T3D σNS and μNS (44) expression plasmids have been described elsewhere. T1L μNS expression plasmid was engineered by amplification of the T1L M3 open reading frame to contain 5′ KpnI and 3′ NotI restriction sites using T1L M3 reverse-genetics plasmid pT7-M3T3D (49) and the following primers: T1L_M3_KpnI5′, CGACGGTACCATGGCTTCATTCAAGGGATTCTCCGTC, and T1L_M3_NotI3′, ATCACAGGCGGCCGCTTACAGCTCATCAGTTGGAACGGAG. The amplified DNA was digested with NotI-HF and KpnI-HF (New England BioLabs [NEB]) and purified from agarose gel fragments following electrophoresis. The purified PCR product was ligated into pcDNA3.1+ vectors between the NotI-HF and KpnI-HF restriction sites.

### Transfections and siRNA silencing assays.

HeLa cells were transfected with mCherry-ER-3 plasmid expressing mCherry fused with calreticulin, ER signal peptide, and KDEL (Addgene) alone or in combination with µNS or σNS plasmids using Trans-IT 2020 (Mirus) as a transfection reagent according to the manufacturer’s instructions. At 24 h posttransfection, cells were fixed with 4% paraformaldehyde (PFA) in phosphate-buffered saline (PBS) at room temperature for 20 min. Cells were permeabilized with 0.25% saponin and imaged using immunofluorescence microscopy.

For silencing experiments, HeLa cells were transfected with mCherry-ER-3 and siRNAs specific for σNS (Dharmacon, sense sequence: UGA UGG ACU UAA GGG AUU AUU), µNS (Dharmacon, sense sequence: GAG CAA GGG UCU AUG UCU AUU), or luciferase (Qiagen, catalog no. 1022073, sense sequence: CUU ACG CUG AGU ACU UCG ATT) using Lipofectamine RNAiMAX (Invitrogen) according to the manufacturer’s instructions. At 24 h posttransfection, virus was adsorbed to HeLa cells at a multiplicity of infection (MOI) of 1 PFU/cell on glass coverslips. Following incubation at 37°C for 24 h, cells were fixed with 4% PFA in PBS at room temperature for 20 min and processed for immunofluorescence.

### BrUTP incorporation assay.

Newly synthesized viral RNA was labeled in reovirus-infected cells using a bromouridine (BrU) incorporation assay. HeLa cells were adsorbed with reovirus at an MOI of 1 PFU/cell. At 14 h postadsorption, cells were incubated for 6 h with 50 µg/ml α-amanitin (Sigma) to block cellular RNA synthesis. At 15 h postinfection, cells were incubated for 5 h with 10 mM BrU (Sigma), washed with PBS, fixed for 1 h with 4% PFA in PBS, and processed for cryosectioning and immunogold labeling using a monoclonal antibromodeoxyuridine antibody (Sigma) diluted 1:50 in saturation buffer for 1 h followed by a secondary antibody conjugated with 10-nm colloidal gold particles for 30 min. Samples were imaged using a JEOL JEM-1011 transmission electron microscope.

### Confocal microscopy.

HeLa cells cultivated on glass coverslips in 6-well plates were adsorbed with reovirus at an MOI of 1 PFU/cell. Following incubation at 37°C for 14 h, cells were fixed with 4% PFA in PBS (pH 7.4) at room temperature for 20 min, permeabilized with 0.25% saponin, and labeled with a calreticulin-specific antibody (Novus Biologicals, Inc.), a PDI-specific antibody (MD-12; Sigma), σNS-specific antibodies 2F5 and VU82 (53), or µNS-specific antibodies VU267 and chicken polyclonal antiserum, provided by John Parker (Cornell University) and previously described (21). 4′,6-Diamidino-2-phenylindole (DAPI; Invitrogen) was used to stain nuclei. Alexa Fluor-conjugated antibodies (Invitrogen) were used as secondary antibodies. Antibodies and DAPI were diluted in saturation buffer (1% bovine serum albumin [BSA] in PBS) as follows: 1:200 for anti-PDI and anti-calreticulin antibodies and DAPI, 1:1,500 for σNS antibody, 1:1,000 for µNS antibody, and 1:500 for secondary antibodies. Images were acquired using a Leica TCS SP5 confocal microscope.

### STED superresolution microscopy.

HeLa cells were adsorbed with reovirus at an MOI of 1 PFU/cell on glass coverslips. Following incubation at 37°C for 26 h, cells were fixed with 4% PFA in PBS at room temperature for 20 min. Cells were permeabilized with 0.25% saponin and labeled with a σNS-specific antibody and a calreticulin-specific antibody followed by secondary antibodies conjugated with Alexa Fluor 488 and 546 (Invitrogen). Images were acquired using a Leica TCS SP8 microscope with a 3× STED module for superresolution.

### 3D image reconstructions from serial sections.

HeLa cells were adsorbed with reovirus at an MOI of 1 PFU/cell. Following incubation at 37°C for 14 h, cells were fixed with a mixture of 4% PFA and 1% glutaraldehyde in PBS at room temperature for 1 h, postfixed with 1% osmium tetroxide, dehydrated in increasing concentrations of acetone, and embedded in EML-812 epoxy resin (TAAB Laboratories). Samples were polymerized at 60°C for 48 h. Consecutive ultrathin (~60- to 70-nm) sections were collected on Formvar-coated copper slot grids (TAAB Laboratories), stained, and imaged using a JEOL JEM-1011 transmission electron microscope operating at 100 kV. Three series of 15 consecutive sections were obtained, and the one with the best contrast was processed for 3D reconstruction as described previously (22). Images of reovirus inclusions were obtained using a charge-coupled device (CCD) camera (Gatan) at a nominal magnification of ×40,000 and a resolution of 72 pixels per inch (ppi). Digital images with an 8.82-nm final pixel size were aligned with a free editor for serial section microscopy, Reconstruct (54) (http://synapseweb.clm.utexas.edu/software-0). Segmentation and 3D visualization were conducted using Amira. Movies from the 3D reconstructions were assembled using the Camera Rotate and Movie Maker applications of the Amira software.

### Immunogold labeling of Tokuyasu cryosections.

Cells were fixed with 4% PFA and 0.1% glutaraldehyde in 0.4 M HEPES buffer, pH 7.4, at room temperature (RT) for 2 h. Free aldehyde groups were quenched with 50 mM NH_4_Cl. Cells were removed from the plastic with a rubber policeman and collected by centrifugation in a 1.5-ml Eppendorf tube. The pellet was embedded in 12% gelatin (TAAB Laboratories) in PBS, and after solidification, cubes of 1 mm^3^ were cut and infiltrated with 2.3 M sucrose in PBS at 4°C overnight. Cubes were mounted on metal pins and frozen in liquid nitrogen. Thin cryosections were prepared at −120°C using an FC6 cryoultramicrotome (Leica Microsystems), collected from the diamond knife with a 1:1 mixture of 2% methylcellulose in H_2_O and 2.1 M sucrose in PBS, and placed after thawing on 200-mesh grids with a carbon-coated Formvar film. For single and double immunogold labeling, sections were incubated with primary and secondary antibodies. Primary antibodies were diluted in saturation buffer (1% BSA in PBS) as follows: 1:200 for anti-PDI and anti-calreticulin, 1:50 for anti-dsRNA (English and Scientific Consulting) and anti-BrU (Sigma), 1:100 for rabbit polyclonal λ3-specific antiserum (52), and 1/200 for the affinity-purified rabbit polyclonal anti-KDEL-R (55) provided by Irina Majoul (MPI for Biophysical Chemistry, Göttingen, Germany). Grids were incubated at RT for 1 h. Secondary antibodies conjugated with 10- or 15-nm colloidal gold particles were diluted 1:50 in saturation buffer, and samples were incubated at RT for 30 min. Protein A conjugated with 10-nm colloidal gold particles was diluted 1:100 in saturation buffer. All colloidal gold conjugates were supplied by British Biocell Int. (BBI). After labeling, images were collected using a JEOL JEM-1011 transmission electron microscope operating at 100 kV. At least two independent labeling assays were performed for each experimental condition.

### Electron tomography of Tokuyasu cryosections.

Semithick (~300-nm) Tokuyasu cryosections of reovirus-infected cells were collected on copper grids with parallel bars. Four single-axis tilt series were obtained automatically between −63° and +60° with an angular interval of 1.5°. Images were recorded on an Eagle 4k-by-4k slow-scan charge-coupled device (FEI, Eindhoven, The Netherlands) using FEI software and a Tecnai G2 microscope (FEI) operating at 200 kV. Images were aligned, and tomograms were reconstructed using the IMOD software package (56). The tomogram with best contrast was segmented and processed for 3D visualization with Amira. Tomograms were subjected to noise filtering and automated segmentation to visualize membranes (57).

### METTEM.

To visualize metallothionein-tagged μNS protein molecules, mCherry-T1LM3 230MT-HeLa CCL2 cells were incubated *in vivo* with 0.5 mM HAuCl_4_ (Sigma-Aldrich) in DMEM at 37°C for 15 min. This treatment allows gold nanoclusters to form on metallothionein-tagged proteins (58). Cells were washed with DMEM, fixed in a mixture of 4% paraformaldehyde and 1% glutaraldehyde in 0.4 M HEPES buffer (pH 7.4), washed with deionized water, and incubated for 10 min with silver salts (HQ Silver; Nanoprobes) (33). After washing with deionized water, samples were postfixed and embedded in epoxy resin as described above. Ultrathin sections were stained with uranyl acetate and lead citrate and imaged by TEM. For confirmation of labeling specificity, control HeLa cells (lacking MT-tagged proteins) were incubated with gold and silver and processed as described above. At least three different resin blocks were sectioned for each experimental condition.

### Live-cell imaging.

HeLa cells transfected with mCherry-ER-3 plasmid expressing mCherry fused with calreticulin, ER signal peptide, and KDEL (Addgene) were cultivated on glass-bottom culture p35 plates (Ibidi). At 24 h posttransfection, cells were adsorbed with reovirus strain T1L. From 4 to 10 h postadsorption, fluorescence and differential interference contrast (DIC) images were collected every 15 min using a Leica DMI6000B fluorescence microscope and LAS X software. To identify infected cells, immediately after video recording, samples were processed for immunofluorescence staining using a σNS-specific antibody (2F5) and a secondary antibody conjugated with Alexa 488. U-2 OS mCherry-KDEL cells were transfected with a plasmid encoding residues 1 to 230 of the μNS protein N terminally fused to GFP. At 24 h posttransfection, cells were adsorbed with reovirus strain T1L. From 9 to 18 h postinfection, fluorescence images were collected every 30 min using a Zeiss LSM710 confocal microscope and Zen software.

### CLEM.

HeLa cells engineered to stably express mCherry-μNS were cultured on photoetched grid p35 plates (Ibidi). Cells were adsorbed with reovirus T1L M1-P208S at an MOI of 1 PFU/cell, incubated for 14 h, and imaged using bright-field and fluorescence microscopy. Cells with interesting features were selected using a Leica TCS SP5 confocal microscope and fixed with a mixture of 4% PFA and 0.1% glutaraldehyde in PBS. After fixation and dehydration with ethanol, cells were embedded in EML-812. Resin-embedded cell monolayers were separated from grid plates by immersion in liquid nitrogen and warm water. Preselected cells were localized in the first ultrathin sections that were collected on Formvar-coated 50-GP copper slot grids and stained with uranyl acetate and lead citrate. Three independent CLEM experiments were conducted. A total of 17 areas were selected by confocal microscopy, and three were processed for embedding in epoxy resin and ultramicrotomy. Nine cells were processed by oriented serial sectioning and studied by TEM.

### Quantitative confocal microscopy.

Quantifications were conducted using mock-infected cells (*n =* 166), reovirus-infected cells (*n =* 97), cells transfected with σNS and μNS (*n =* 134), cells transfected with σNS alone (*n =* 147), and cells transfected with μNS alone (*n =* 135). ER remodeling was classified in four categories: (i) branched thin tubules, (ii) unbranched thin tubules, (iii) fragmented, and (iv) collapsed. The numbers of cells with normal ER and cells with zones of ≥15 µm^2^ with low density of labeled ER elements (termed “ER-free zones”) were also quantified. Images were obtained using a Leica TCS SP5 confocal microscope at a magnification of ×63 or ×100 using LAS X software.
