# Supplementary figures and images for: Reovirus σNS and μNS Proteins Remodel the Endoplasmic Reticulum to Build Replication Neo-Organelles
Source: mBio. 2018 Aug 7;9(4):e01253-18. doi: 10.1128/mBio.01253-18 (PMC6083906; doi:10.1128/mBio.01253-18)

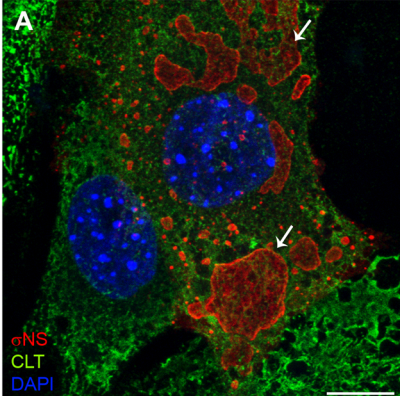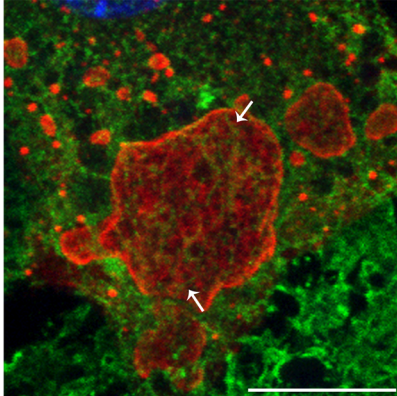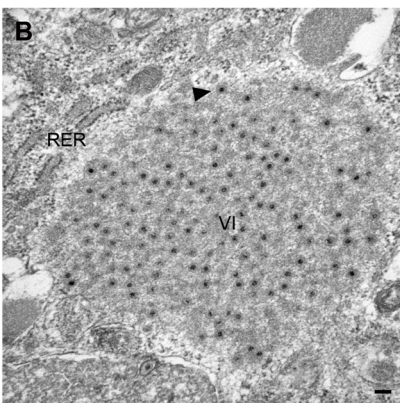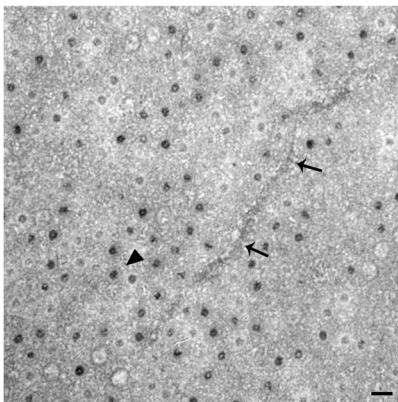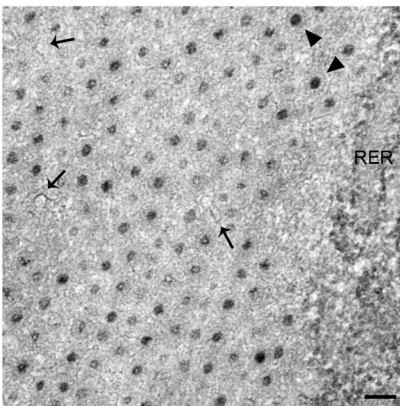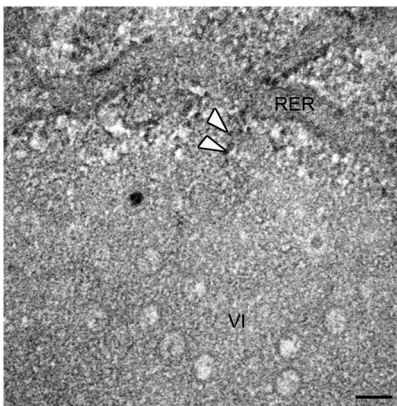

Supplement: FIG S1 [file mbo004184015sf1.pdf]

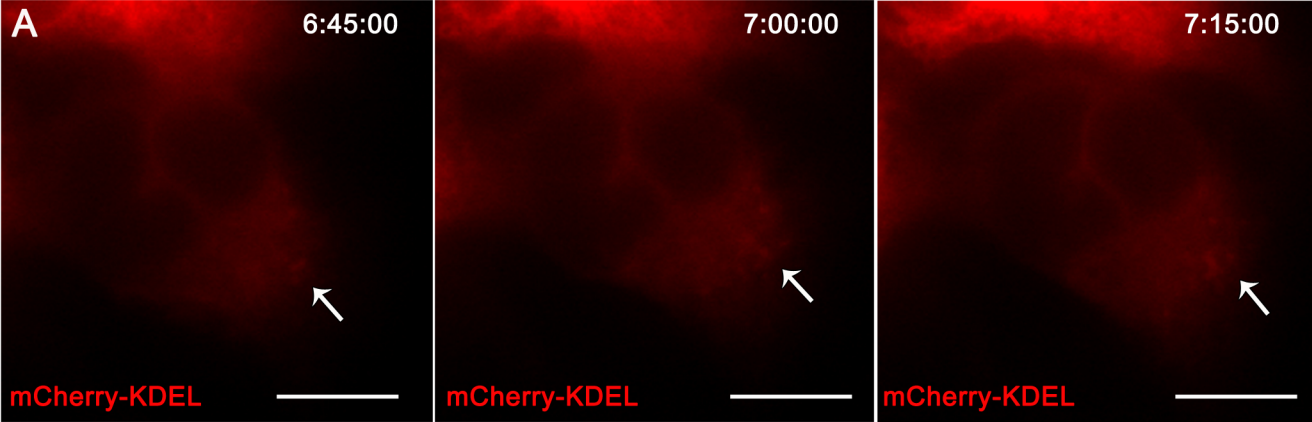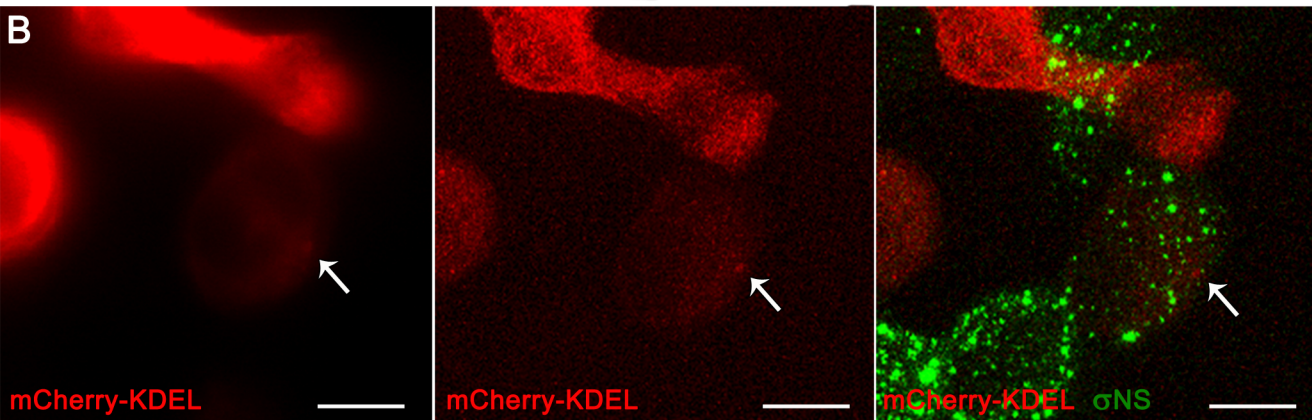

Supplement: FIG S2 [file mbo004184015sf2.pdf]

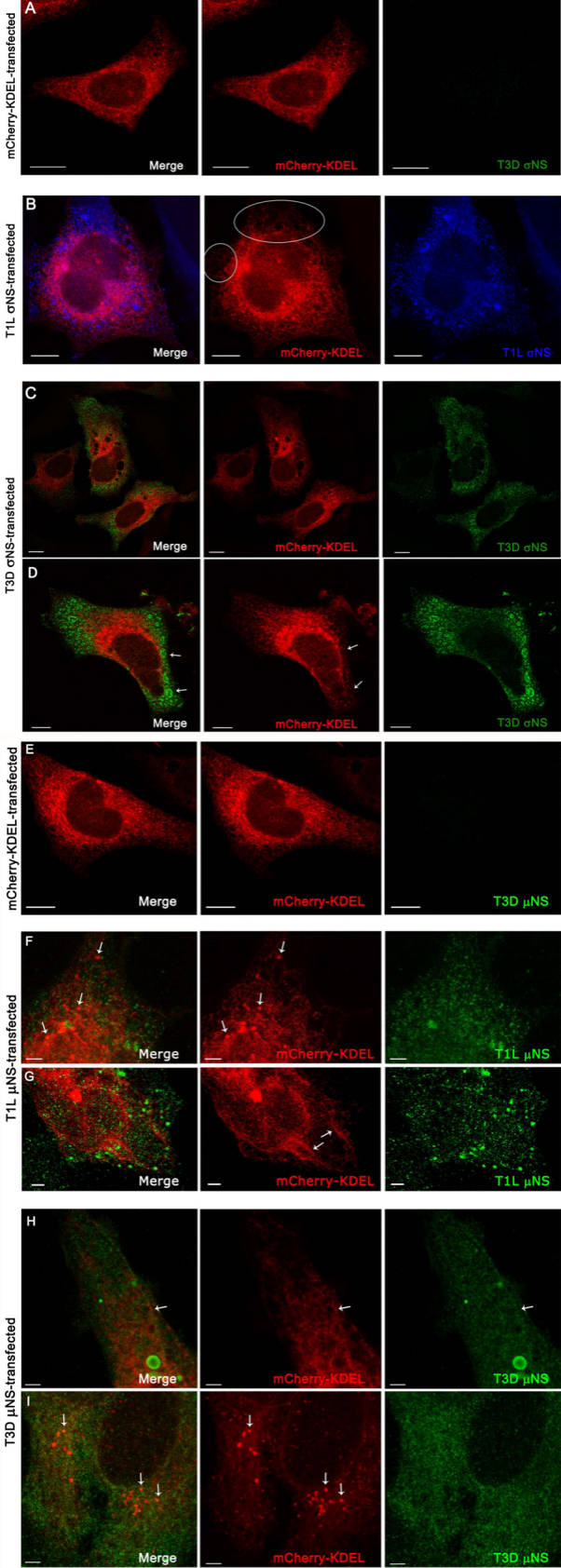

Supplement: FIG S3 [file mbo004184015sf3.pdf]

**Mock**

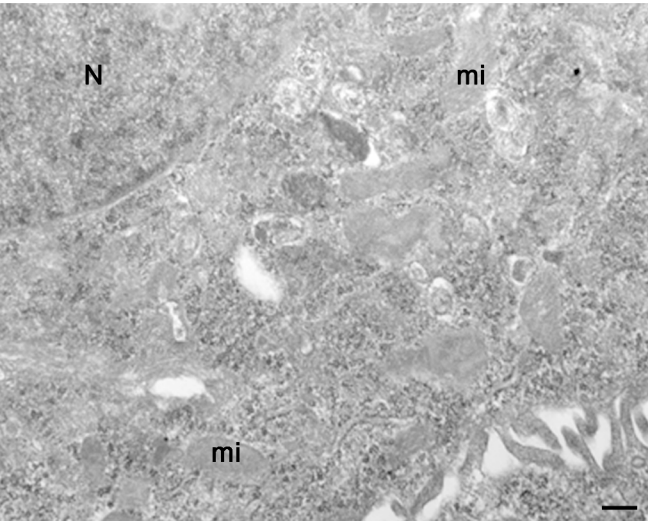

**mCherry- $\mu$ NS-MT + Reovirus**

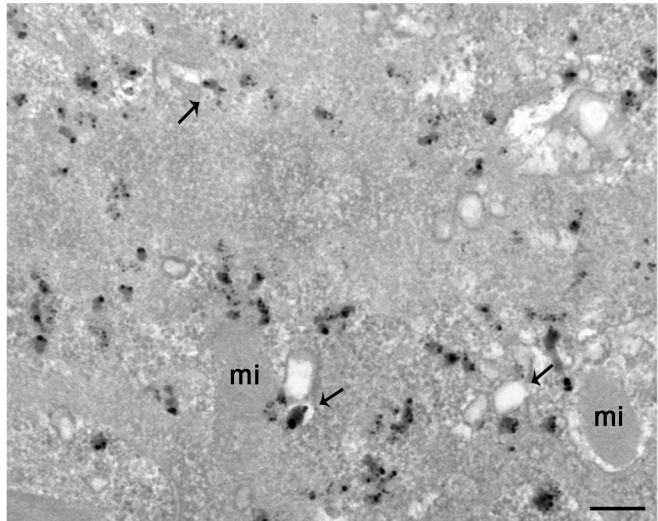

Supplement: FIG S4 [file mbo004184015sf4.pdf]

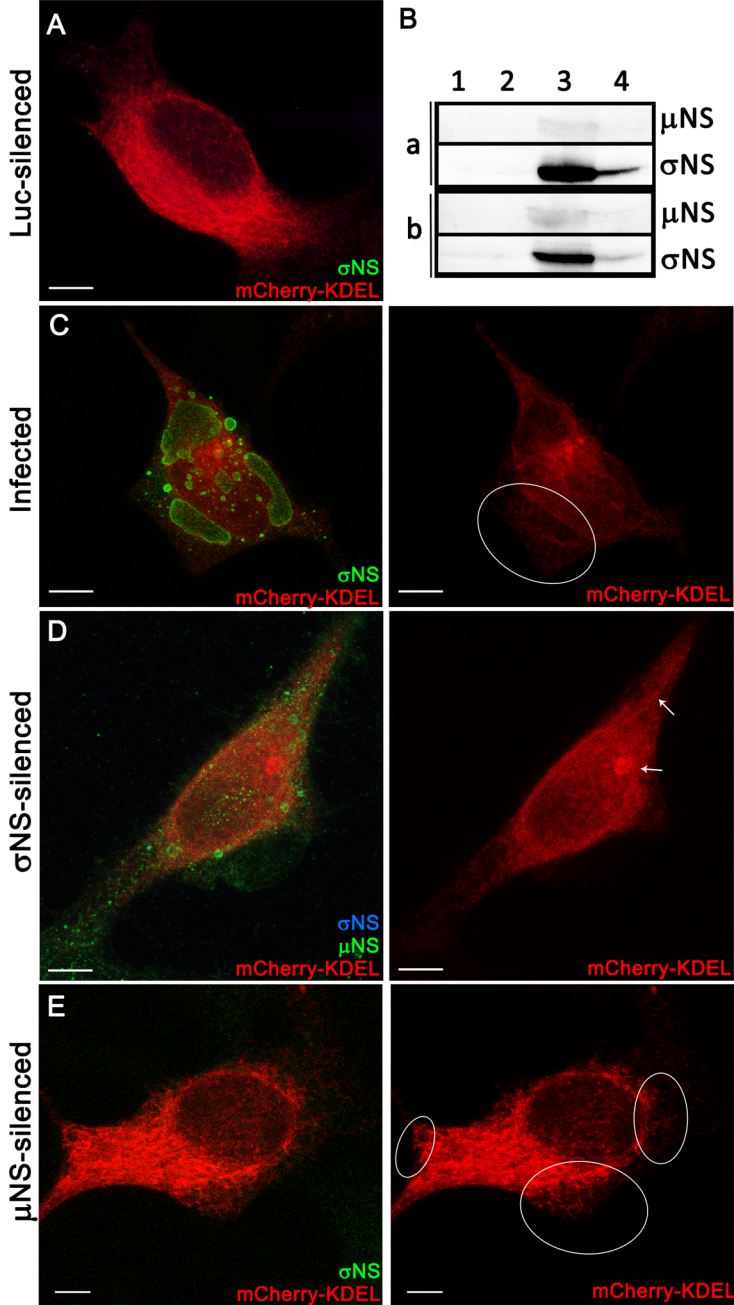

Supplement: FIG S5 [file mbo004184015sf5.pdf]
